# Investigation on *in vitro* dissolution rate enhancement of indomethacin by using a novel carrier sucrose fatty acid ester

**DOI:** 10.1186/1560-8115-20-4

**Published:** 2012-07-19

**Authors:** Songa Ambedkar Sunil, Meka Venkata Srikanth, Nali Sreenivasa Rao, Vengaladasu Raju, Kolapalli Venkata Ramana Murthy

**Affiliations:** 1 A.U. College of Pharmaceutical Sciences, Andhra University, Visakhapatnam, 530003, India

**Keywords:** Solid dispersions, Indomethacin, Sucrose Fatty Acid Ester, Dissolution rate

## Abstract

**Background and the purpose of the study:**

The purpose of the present investigation was to characterize and evaluate solid dispersions (SD) of indomethacin by using a novel carrier sucrose fatty acid ester (SFE 1815) to increase its in vitro drug release and further formulating as a tablet.

**Methods:**

Indomethacin loaded SD were prepared by solvent evaporation and melt granulation technique using SFE 1815 as carrier in 1:0.25, 1:0.5 1:0.75 and 1:1 ratios of drug and carrier. Prepared SD and tablets were subjected to in vitro dissolution studies in 900 mL of pH 7.2 phosphate buffer using apparatus I at 100 rpm. The promising SD were further formulated as tablets using suitable diluent (DCL 21, Avicel PH 102 and pregelatinised starch) to attain the drug release similar to that of SD.. The obtained dissolution data was subjected to kinetic study by fitting the data into various model independent models like zero order, first order, Higuchi, Hixon-Crowell and Peppas equations. Drug and excipient compatibility studies were confirmed by fourier transform infrared spectroscopy, X-ray diffraction, differential scanning calorimetry and scanning electron microscopy.

**Results:**

The in vitro dissolution data exhibited superior release from formulation S_6_ with 1:0.5 drug and carrier ratio using solvent evaporation technique than other SDs prepared at different ratio using solvent evaporation and melt granulation technique. The in vitro drug release was also superior to that of the physical mixtures prepared at same ratio and also superior to SD prepared using common carriers like polyvinyl pyrollidone and PEG 4000 by solvent evaporation technique. Tablets (T_8_) prepared with DCL21 as diluent exhibited superior release than the other tablets. The tablet formulation (T_8_) followed first order release with Non-Fickian release.

**Conclusion:**

SFE 1815 a novel third generation carrier can be used for the preparation of SD for the enhancement of *in vitro* drug release of indomethacin an insoluble drug belonging to BCS class II.

## Introduction

The therapeutic efficacy of a drug product intended to be administered by the oral route depends upon its absorption in the gastro-intestinal tract to end with bioavailability. It is well established that dissolution is recurrently the rate-limiting step in the gastrointestinal absorption of a drug from a solid dosage form which belongs to BCS class II (low soluble and high permeable). The drug release from poorly soluble drugs has been shown to be unpredictable and still remains a problem to the pharmaceutical industry [1]. Several methods that have been employed to improve the solubility of poorly water soluble drugs include increasing the particle surface area available for dissolution by milling [2], improving the wettability with surfactants or doped crystals [3], decreasing crystallinity by preparing a solid dispersion [4], use of inclusion compounds such as cyclodextrin derivatives [5], use of polymorphic forms or solvated compounds [6] and use of salt forms. There are several advantages and disadvantages for the above given methods. Solid dispersions (SD) represent an ideal pharmaceutical technique for increasing the dissolution, absorption and therapeutic efficacy of drugs with poor aqueous solubility. The term “solid dispersion” refers to the dispersion of one or more active ingredients in an inert carrier or matrix in the solid state prepared by melting, solvent, or melting solvent methods [7] which has been used by various researchers who have reported encouraging results with different drugs [8]. The method of preparation and the type of the carrier used are important in influencing the properties of such solid dispersions [9]. Among the carriers used in the formation of solid dispersions, polyethylene glycol and polyvinyl pyrrolidone are the most commonly used. The first generation (urea) and second generation (PEG, polyvinyl pyrrolidone, HPMC, hydroxylpropyl cellulose, starch derivatives, cyclodextrins) of carriers have many disadvantages when compared to the use of third generation carriers (poloxamer, gelucire, sucrose fatty acid esters) which are non-ionic and led to development of superior solid dispersions.

Sucrose fatty acid esters (SFE) are nonionic surface active agents which are mono-, di-, and tri-esters of sucrose with fatty acids, manufactured from purified sugar or hydrogenated edible tallow or edible vegetable oils. These consist of sucrose residues as the hydrophilic group or polar head and fatty acid residues as the lipophilic group or non-polar head with a unique emulsification property that tolerates any temperature variation [10,11]. SFE are currently regulated by the U.S. Food and Drug Administration (FDA) as food additives under chapter 21, section 172.859 of the Federal Code of Regulations (CFR). However, studies of SFE in the area of commonly used tablet formulations are limited and emphasize using specific types of SFE for particular approaches. These are non-toxic and biodegradable, as they can be enzymatically hydrolyzed to sucrose and fatty acids prior to intestinal absorption or excreted in faeces, depending on the degree of esterfication with a wide range of HLB values 1 – 16 [12,13].

The present investigation was focused on exploring sucrose fatty acid ester as a drug carrier to increase the drug solubility and the dissolution rate of indomethacin by formation of solid dispersions using various methods. The dissolution characteristics and physicochemical modification of the indomethacin-SFE solid dispersions were investigated by *in vitro* dissolution test, FTIR, XRD, SEM and thermal analysis (DSC). These solid dispersions were formulated into tablets after optimizing with suitable diluent used in the study and were evaluated for physicochemical characterization.

## Experimental

### Materials

Indomethacin was a gift sample from Macleods pharmaceuticals Ltd. India. Sucrose fatty acid ester 1815 was obtained from Mitsubishi-Kagaku Foods Corporation, Japan. Avicel PH 102 was obtained from FMC Biopolymer. DCL 21 was purchased from Zeel Pharmaceuticals, India. All other chemicals were of reagent grade and used as received.

## Methods

### Composition of solid dispersions

Solid dispersions contained of 1:0.25, 1:0.5, 1:0.75 and 1:1 of indomethacin and SFE 1815 prepared by melt granulation and solvent evaporation methods. Physical mixtures were prepared only for the promising ratio for comparision. Table 1 lists the solid dispersions prepared along with the method employed for preparation, composition and codes.

**Table 1:** Composition of different SD using SFE 1815

Code name	Method	Polymer	Ratio (Drug: polymer)
S_1_	Melt Granulation	SFE 1815	1:0.25
S_2_			1:0.5
S_3_			1:0.75
S_4_			1:1
S_5_	Solvent Evaporation	SFE 1815	1:0.25
S_6_			1:0.5
S_7_			1:0.75
S_8_			1:1
S_9_	Physical Mixing	SFE 1815	1:0.5
S_10_	Solvent Evaporation	PVP	1:0.5
S_11_	Solvent Evaporation	PEG 4000	1:0.5

### Preparation of solid dispersions

#### Melt granulation method

Accurately weighed amounts of carrier were placed in an aluminum pan on a hot plate and melted, with constant stirring, at a temperature of about 50 °C. An accurately weighed amount of indomethacin was incorporated into the melted carrier with stirring to ensure homogeneity. The mixture was heated until a clear homogeneous melt was obtained. The pan was then removed from the hot plate and allowed to cool at room temperature and the obtained damp mass is passed through sieve no #40. The granules obtained were transferred to a polybag and stored in desiccator for further studies.

#### Solvent evaporation method

Accurately weighed amounts of indomethacin and carrier (SFE 1815) were dissolved in minimum quantities of methanol in a china dish. The solution was stirred till slurry was formed. The solvent was evaporated under reduced pressure at 40 °C, and the resulting residue was dried under vacuum for 3 h, stored in a desiccator at least overnight, ground in a mortar, and passed through mesh no #40.

#### Physical mixtures

Physical mixtures were obtained by pulverizing accurately weighed amounts of drug and polymer in a glass mortar and carefully mixed until a homogeneous mixture was obtained. Drug and carrier ratio of 1:0.5 were prepared and subsequently stored at room temperature in desiccator.

#### Preparation of tablets

SD powder, diluents, disintegrant and binder were weighed as per formulae given in Table 2, these were then passed through sieve no # 40, transferred to a poly bag and blended for 5 min. To this homogeneous blend, magnesium stearate presifted through # 60 was added and blended for 2 min. The resulting blend was compressed on Cadmach 16 station compression machine under a common compression force of 2-3 Kg/cm^2^, using 6 mm, round, flat faced punches.

**Table 2:** Formulae of tablets

Formulation	T_1_	T_2_	T_3_	T_4_	T_5_	T_6_	T_7_	T_8_	T_9_
Indo + SD	37.5	37.5	37.5	37.5	37.5	37.5	37.5	37.5	37.5
Pregelatinised Starch	25	55	75						
Avicel PH 102				25	55	75			
DCL 21							25	55	75
Crocarmellose Sodium	3	3	3	3	3	3	3	3	3
PVP K 30	3	3	3	3	3	3	3	3	3
Magnesium Stearate	1.5	1.5	1.5	1.5	1.5	1.5	1.5	1.5	1.5

#### In vitro dissolution studies

Powder equivalent to indomethacin 25 mg for SD and tablets were introduced into dissolution medium. The dissolution medium is 900 mL of phosphate buffer pH 7.2, rotational speed of the basket was set at 100 rpm at 37 ± 0.5 °C. Aliquots (5 ml each) were withdrawn at predetermined time intervals by means of a syringe fitted with a 0.45 μm pre-filter and immediately replaced with 5 mL of fresh medium maintained at 37 ± 0.5 °C. The samples were analyzed for indomethacin using U.V. double beam Elico SL 210 model at 318 nm. For comparison, dissolution studies of pure indomethacin and INDOCAP marketed capsules along with PM and SD prepared with polyvinyl pyrollidone (PVP) and PEG 4000 at drug and polymer ratio of 1:0.5 employing solvent evaporation technique were also performed. All the dissolution experiments were carried out in triplicate. Comparison of dissolution profiles was done to quantify the difference in rate and extent of drug release as influenced by the formulation and process variables in order to find out the mode of drug release and their kinetics.

#### Release kinetics

As a model-dependent approach, the dissolution data was fitted to five popular release models such as zero-order, first-order, Higuchi [14], Hixon-Crowel [15] and Korsmeyer -peppas equations. The order of drug release from matrix systems was described by using zero order kinetics or first orders kinetics. The mechanism of drug release from matrix systems was studied by using Higuchi and Hixon-Crowel equation. Model with the highest coefficient correlation (r) was judged to be a more appropriate model for the dissolution data.

According to Korsmeyer-Peppas equation, the release exponent n value is used to characterize different release mechanisms. If the n value is 0.5, the release mechanism follows Fickian diffusion. If n value is >0.45 or <0.89, the mechanism follows non-Fickian (anomalous) diffusion and when n = 0.89 it will be non-Fickian case II and if n > 0.89 it will be non-Fickian super case II transport [16]. The equations for different models are represented in Table 3.

**Table 3:** Mathematical models for comparison of dissolution profiles

Model	Equation
Zero-order	$Q_{t}=Q_{0}+K_{0}$
First-order	${\mathrm{InQ}}_{t}={\text{InQ}}_{0-}K_{1}t$
Higuchi	$Q_{t}{\text{=K}}_{H}\sqrt{t}$
Hixon-Crowell	$Q_{r}^{1/3}-Q_{t}^{1/3}=K_{s}t$
Korsmeyer-Peppas	QtQ∞=Kktn

Q_t_: amount of drug released in time t, Q_0_: initial amount of drug in the Tablet, Q_r:_ remaining amount of the drug in tablet, Q_∞_: fraction of drug released at time t, K_o_, K_1,_ K_H_, K_s_, K_K –_ Rate order constants.

#### Fourier transform infrared spectroscopy

FTIR spectra can be used to detect drug excipient interactions by following the shift in vibrational or stretching bands of key functional groups. FTIR spectra were obtained by using Alpha FTIR spectrophotometer (Bruker Optik GmbH, Germany). All the spectra were analyzed using OPUS 6.5 software. Samples were prepared by KBr pellet method, which had been prepared by gently mixing 1 mg of the sample with 200 mg of KBr. The spectra were scanned over a wave number range of 4000 - 500 cm^−1^.

#### X-ray diffraction

The physical state of indomethacin in different samples was evaluated with X-ray powder diffraction. XRD is a powerful tool in detecting crystallinity. The X-ray diffraction patterns were recorded on X-Ray diffractometer (PW 1729, Philips, Netherlands). XRD patterns were recorded using monochromatic Cu Kα radiation with Nitrogen filter at a voltage of 40 keV and a current of 40 mA. The sample was analyzed over 2θ range of 5-30° and the data was processed with Diffrac Plus V1.01 software.

#### Differential scanning calorimetry

DSC is a frequently used thermo analytical technique that generates data on melting endotherms and glass transitions. DSC was performed utilizing Mettler DSC 821 (Mettler-Toledo, Switzerland). Samples of 3-4 mg were encapsulated and hermetically sealed in flat bottomed aluminum pan with crimped on lid. Samples were allowed to equilibrate for 1 min and then heated in a nitrogen atmosphere over a temperature range from 25 °C to 240 °C with a heating rate of 5 °C/min. An empty aluminum pan is served as reference. Nitrogen was used as a purge gas, at the flow rate of 20 mL/min for all the studies. Reproducibility was checked by running the sample in triplicate. Thermograms were obtained by the STAR^e^ SW 9.10 software and reported.

#### Scanning electron microscopy

SEM has been employed to study the morphology of the samples. The samples were mounted on the SEM sample stab, using a double sided sticking tape and coated with gold (200 A°) under reduced pressure (0.001 torr) for 5 min using an ion sputtering device (Jeol JFC-1100 E, Japan). The gold coated samples were observed under the SEM (JEOL JSM-840A, Japan) and photomicrographs of suitable magnifications were obtained with the aid of a software system (LINK^ISIS^, Oxford, UK).

## Results and discussion

### In vitro dissolution studies

#### Solid dispersions

**Figure 1:**
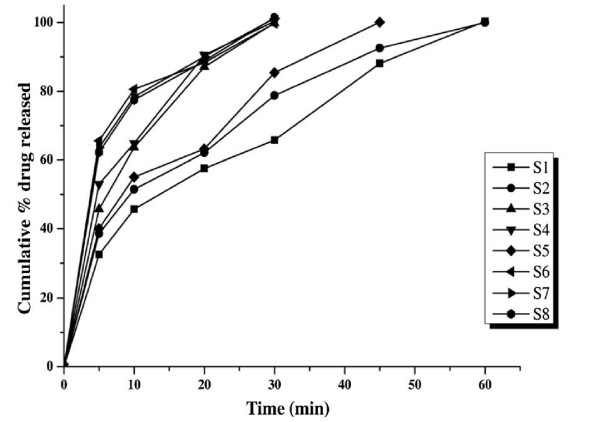
**Dissolution profiles of solid dispersions with SFE 1815 as carrier, S**_**1**_**–S**_**4**_**(melt granulation), S**_**5**_**–S**_**8**_**(solvent evaporation).**

**Figure 2:**
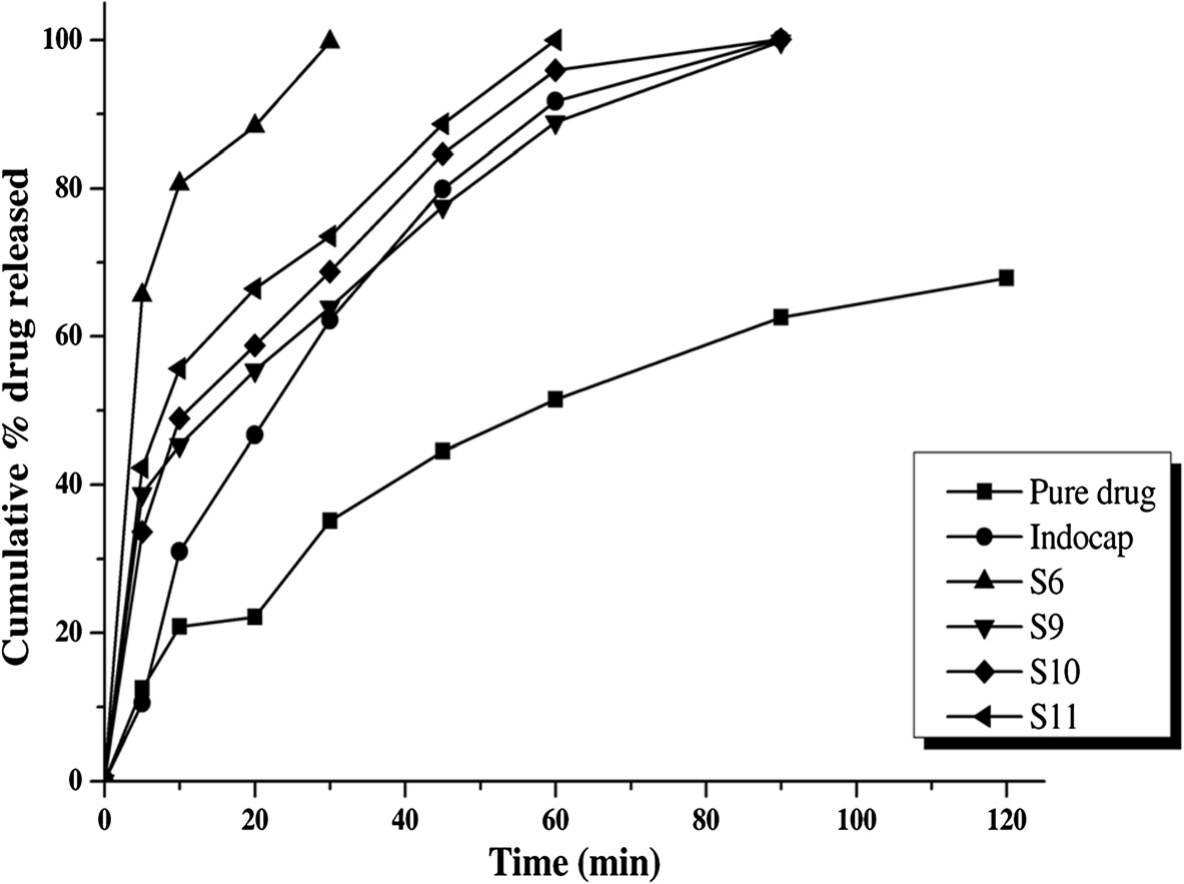
**Comparative dissolution profiles of solid dispersions S**_**6**_**(SFE 1815), S**_**9**_**(physical mixtures), S**_**10**_**(PVP), S**_**11**_**(PEG 4000) along with pure drug and marketed capsule (Indocap).**

Gradual increase in drug release of the prepared SD was observed with increase in concentration of the polymer up to an extent, further increase in concentration did not increase the drug release. Maximum drug release was obtained at the end of 30 min for S_6_ by solvent evaporation technique using SFE 1815 polymer in 1:0.5 drug and polymer ratio. Whereas using the same polymer but employing melt granulation technique gave less drug release. The enhanced drug releases from SD prepared with solvent evaporation technique in this study are in co-relation with the previous study conducted by Patel et al. on SD prepared using PEG 6000 and PVP using solvent evaporation and melting methods [17]. Only 35.10%, 63.90%, 68.78% and 73.54% drug release was observed form pure drug, S_9_ (PM), SD using PVP (S_10_) and PEG 4000 (S_11_) respectively in 30 min, whereas 99.77% drug was released from S_6_ which is shown in Figure 1. Earlier workers also tried out to enhance the dissolution of indomethacin by SD technique, El-Badry et al. prepared SD using PEG4000 and Gelucire 50/13 using hot melting method, results have shown that more amount of carrier was required and also 90 min was required for complete release of drug [18]. The capability to enhance or increase the drug release and bioavailability of the insoluble drug by SFE 1815 depends upon the common factors like excellent wettability, which could be observed clearly from the solid dispersion since it rapidly left the surface and was dispersed in the bulk of the dissolution medium which markedly increased indomethacin solubility and also specific features like (i) HLB value – higher the HLB value greater is the ability to enhance (ii) length of fatty acid chain - shorter fatty acid increases the release more than the longer fatty acids [19] (iii) number of carbon atoms in the fatty acid chain [19] (v) proportion of monoesters –higher the proportion of monoesters higher is the hydrophilicity of the surfactant [12,20,21]. The main reason for better drug release of SD using SFE 1815 is the HLB value which is 15 and the number of monoesters (70%) in the ester composition of the carrier. The comparative dissolution profiles of S_6_ with SD prepared with other carriers, PM prepared with same ratio as S_6_, pure drug and marketed capsule are given in Figure 2.

It is well known from the literature and practical knowledge that the SD are unstable as such, but stable when formulated as a tablet dosage form. The best SD (S_6_) prepared by solvent evaporation technique in 1:0.5 of drug: SFE1815 ratio was selected for the development of tablets, which gave a superior and enhanced release profile than SD prepared by melt granulation using same carrier, SD prepared by common carriers by solvent evaporation technique and PM.

**Figure 3:**
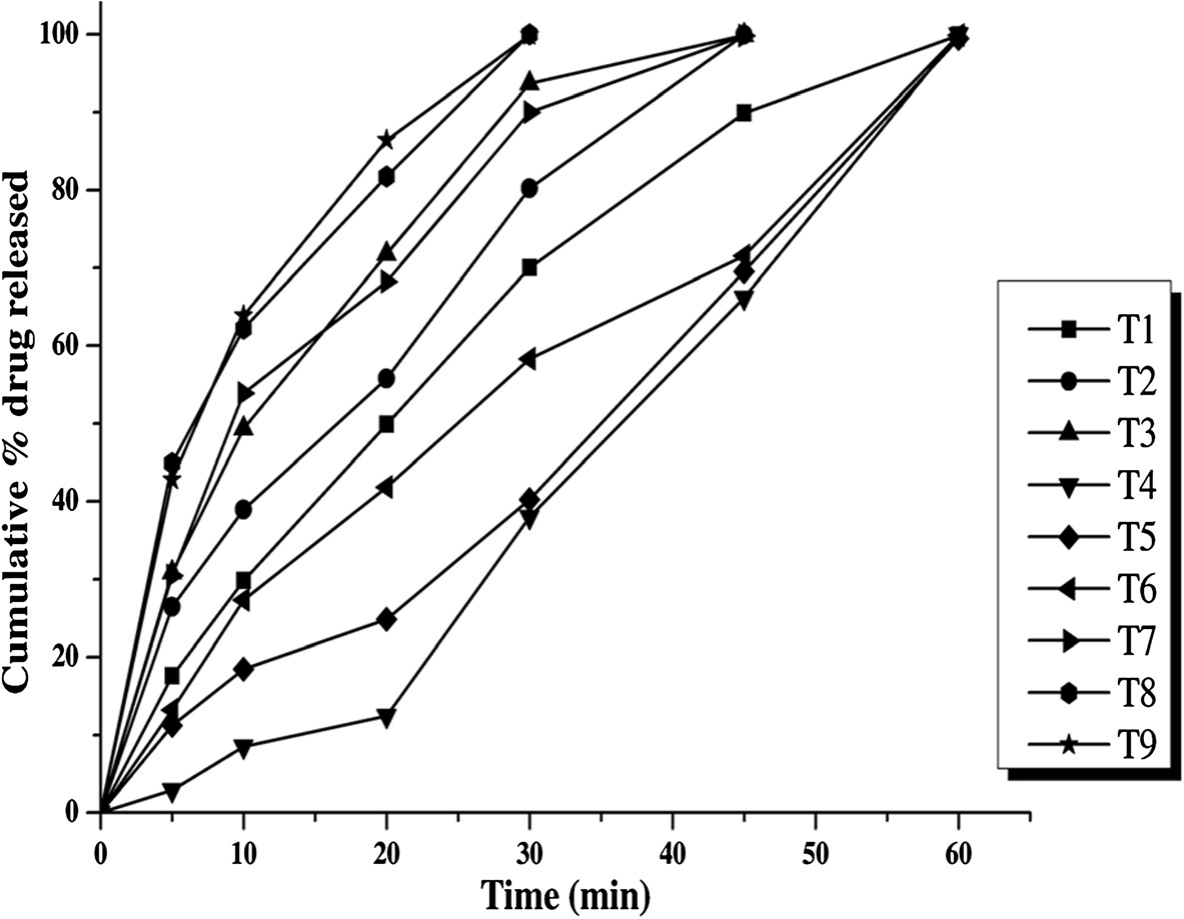
**Dissolution profiles of tablets prepared with different diluents A) T**_**1-3**_**(pregelatinised starch), T**_**4-6**_**(Avicel PH102) and T**_**7-9**_**(DCL 21).**

Tablets were developed using different diluents (pregelatinised starch, Avicel PH102 and DLC 21) in different concentrations (25%, 55% and 75%). More than 99% of the drug was released from all the formulations. T_8_ formulation with 55% w/w of DCL21 gave maximum drug release in 30 min. The initial lag in drug release when compared with SD was due to the disintegration time required for the tablet and the profiles are shown in Figure 3.

Even though pregelatinised starch is more soluble than Avicel PH102 the percent of drug released from the tablets was less when compared to Avicel PH102. The possible reason for that release could be the swelling nature of pregelatinsed starch [22]. As the concentration of the pregelatinized starch was increased, the percent of drug release decreased, which is one of the reason for its use in the development of sustained release tablets [23]. Tablets prepared with DCL 21 as diluent gave better release than those prepared with Avicel PH 102 due to the more hydrophilic nature and solubility of DCL 21.

#### Release kinetics

The drug release of indomethacin from the formulations T_2_ and T_3_ followed zero order kinetics which was indicated by higher ‘r’ values of zero order release model. T_1_, T_4_, T_5_, T_6_, T_7_, T_8_, and T_9_ followed first order release model which was indicated by the higher ‘r’ value.

The relative contributions of drug diffusion and erosion to drug release were further confirmed by subjecting the dissolution data to Higuchi model and Hixon Crowell model. It was found that T_2_ and T_3_ followed zero order kinetics with Non-Fickian diffusion mechanism. T_1_, T_4_, T_5_, T_7_, T_8_ and T_9_ followed first order with Non-Fickian diffusion mechanism. T_6_ formulation followed first order release with erosion mechanism as the ‘r’ value obtained is greater for Hixon Crowell mechanism. The promising tablet formulation T_8_ followed first order release with Non-Fickian diffusion mechanism. Results of various order plots for the tablets are shown in Table 4.

**Table 4:** Kinetic models of core tablets

Model	Zero Order		First order		Higuchi	Hixon-Crowel	Pepas	
Batch	R	K_0_	r	K	r	r	r	n
T_1_	0.990	1.56	0.998	0.028	0.979	0.928	0.995	0.771
T_2_	0.991	1.587	0.958	0.024	0.931	0.915	0.979	0.858
T_3_	0.982	1.687	0.944	0.023	0.893	0.888	0.986	0.881
T_4_	0.977	1.66	0.984	0.049	0.990	0.985	0.997	0.719
T_5_	0.979	2.10	0.983	0.051	0.992	0.968	0.996	0.610
T_6_	0.938	2.13	0.976	0.087	0.993	0.997	0.991	0.552
T_7_	0.937	2.07	0.982	0.072	0.994	0.993	0.984	0.532
T_8_	0.935	2.97	0.992	0.083	0.998	0.973	0.999	0.560
T_9_	0.934	3.05	0.999	0.099	0.998	0.990	0.995	0.573

#### Fourier transform infrared spectroscopy

Pure indomethacin spectra showed characteristic peaks at 3020 cm^–1^ (aromatic C-H stretching), 2965 cm^–1^ (C-H stretching vibrations), 1761 cm^–1^ (C = O stretching vibrations), 1261 cm^–1^ (asymmetric aromatic O-C stretching), 1086 cm^−1^ (symmetric aromatic O-H stretching).

**Figure 4:**
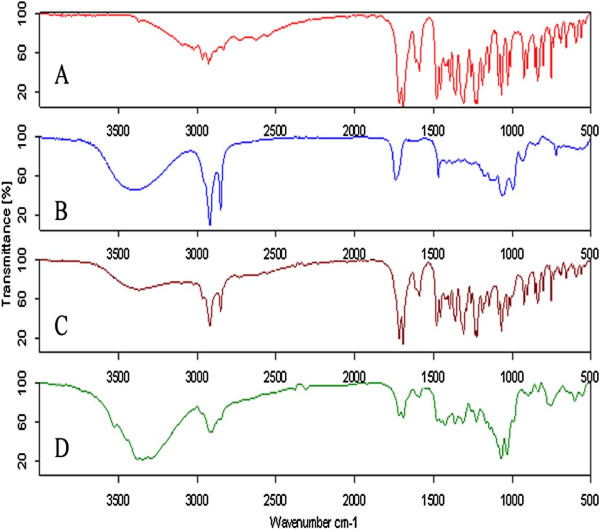
**FTIR spectra of A) indomethacin, B) SFE 1815, C) S**_**6**_**and D) T**_**8**_.

SD (S_6_) and tablet formulation (T_8_) also exhibited the characteristic peaks of indomethacin with no additional peaks observed in the spectra, indicating retention of chemical identity of indomethacin as shown in Figure 4. However, intensity of peaks corresponding to the drug was reduced or broadened in the SD and tablet formulations, possibly due to the mixing with the surfactant and addition of other excipients. The FTIR spectra data confirmed that SFE 1815 did not alter the performance characteristics indicating their compatibility of the drug.

#### X-ray diffraction

**Figure 5:**
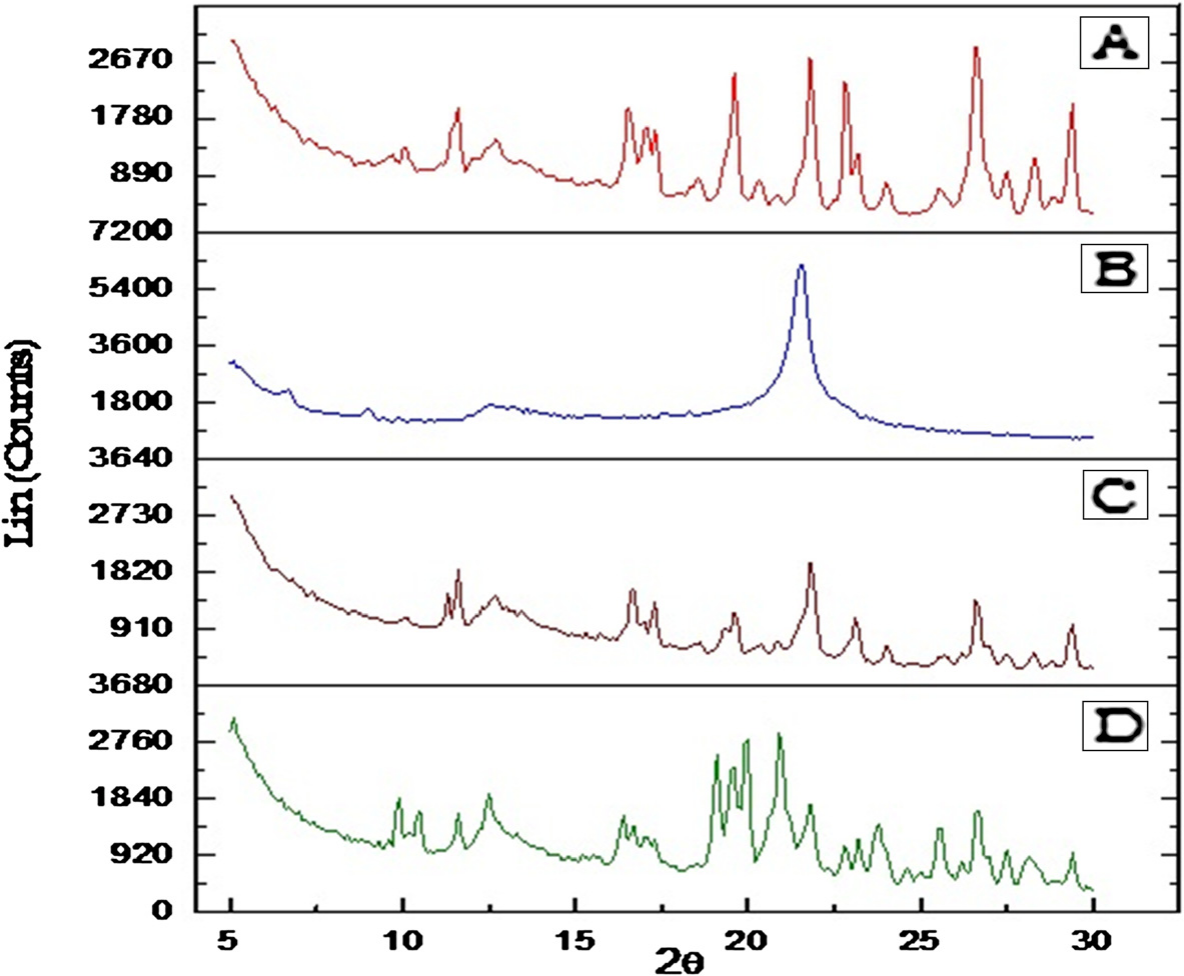
**X-ray diffractograms of A) Indomethacin, B) SFE 1815 C) S**_**6**_**and D) T**_**8**_.

The X-ray diffractograms of pure drug indomethacin and promising formulations are shown in Figure 5. The diffractogram of indomethacin showed characteristic sharp intensity diffraction peaks at 2θ values of 11.51°, 12.76°, 16.62°, 19.54°, 21.84°, 22.78°, 26.64°, 27.47° and 29.35°, which reflected the crystalline nature of drug. Both the formulations (S_6_ and T_8_), showed diffraction peaks at respective 2θ values of pure indomethacin although their relative intensities were reduced or there was slight shift in their peaks, suggesting reduced degree of crystallinity of drug in these formulations.

#### Differential scanning calorimetry

**Figure 6:**
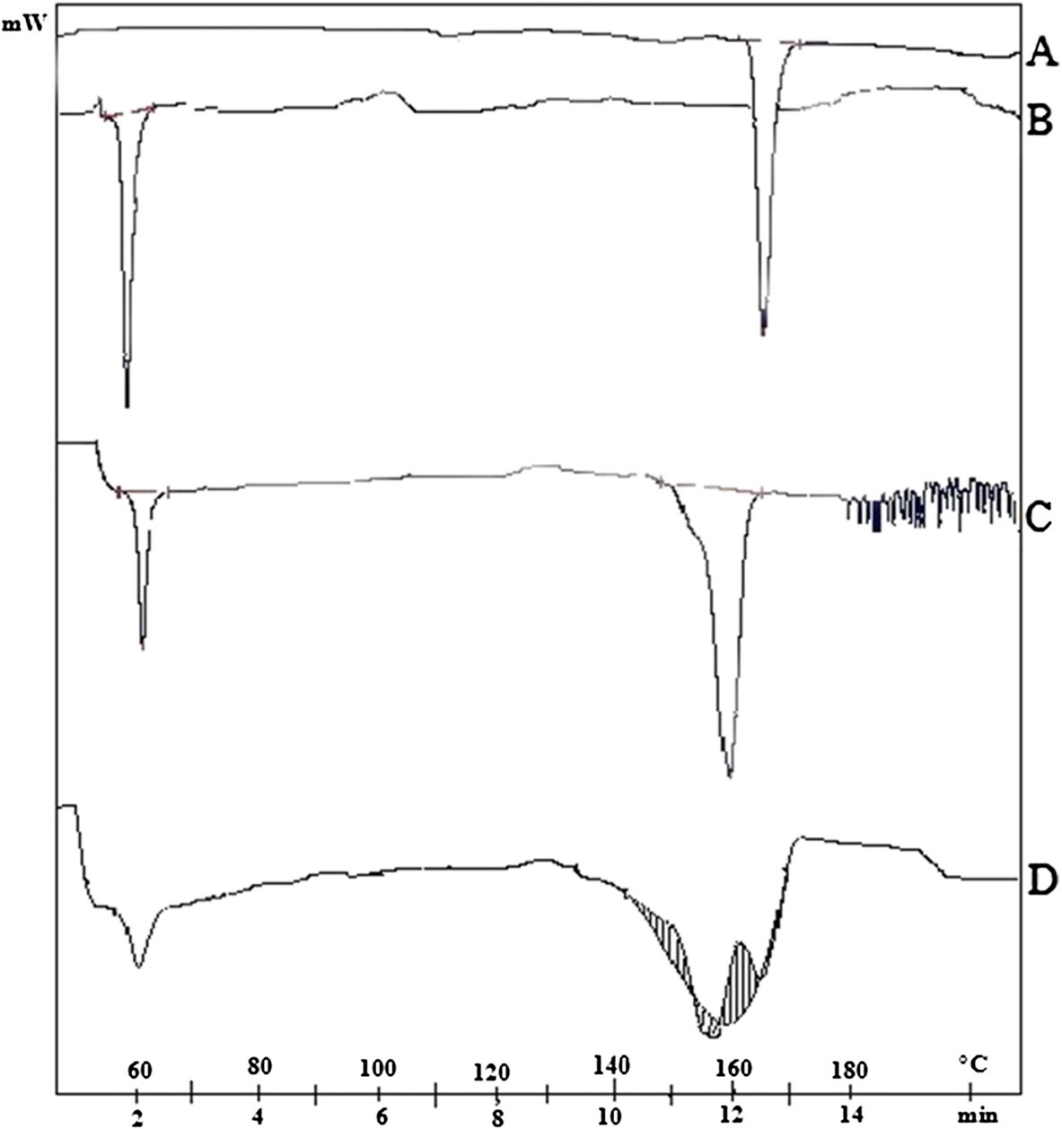
**DSC chromatographs of A) Indomethacin B) SFE 1815 C) S**_**6**_**D) T**_**8**_.

The DSC thermogram of pure indomethacin exhibited a sharp endothermic peak at 164 °C corresponding to its melting point, indicating its crystalline nature. SFE 1815 showed endothermic melting peak at 54.2 °C. There is a shift in the melting peak of indomethacin in SD (S_6_) and tablet (T_8_) to 158.2 °C and 158.4 °C respectively as indicated in Figure 6. The shift observed in the melting peak of indomethacin in the formulations may be due to physical interaction between the drug and excipient. Compared to pure drug the melting peak was broadened to some extent in the formulations which may be due to changes in its crystalline form.

#### Scanning electron microscopy

**Figure 7:**
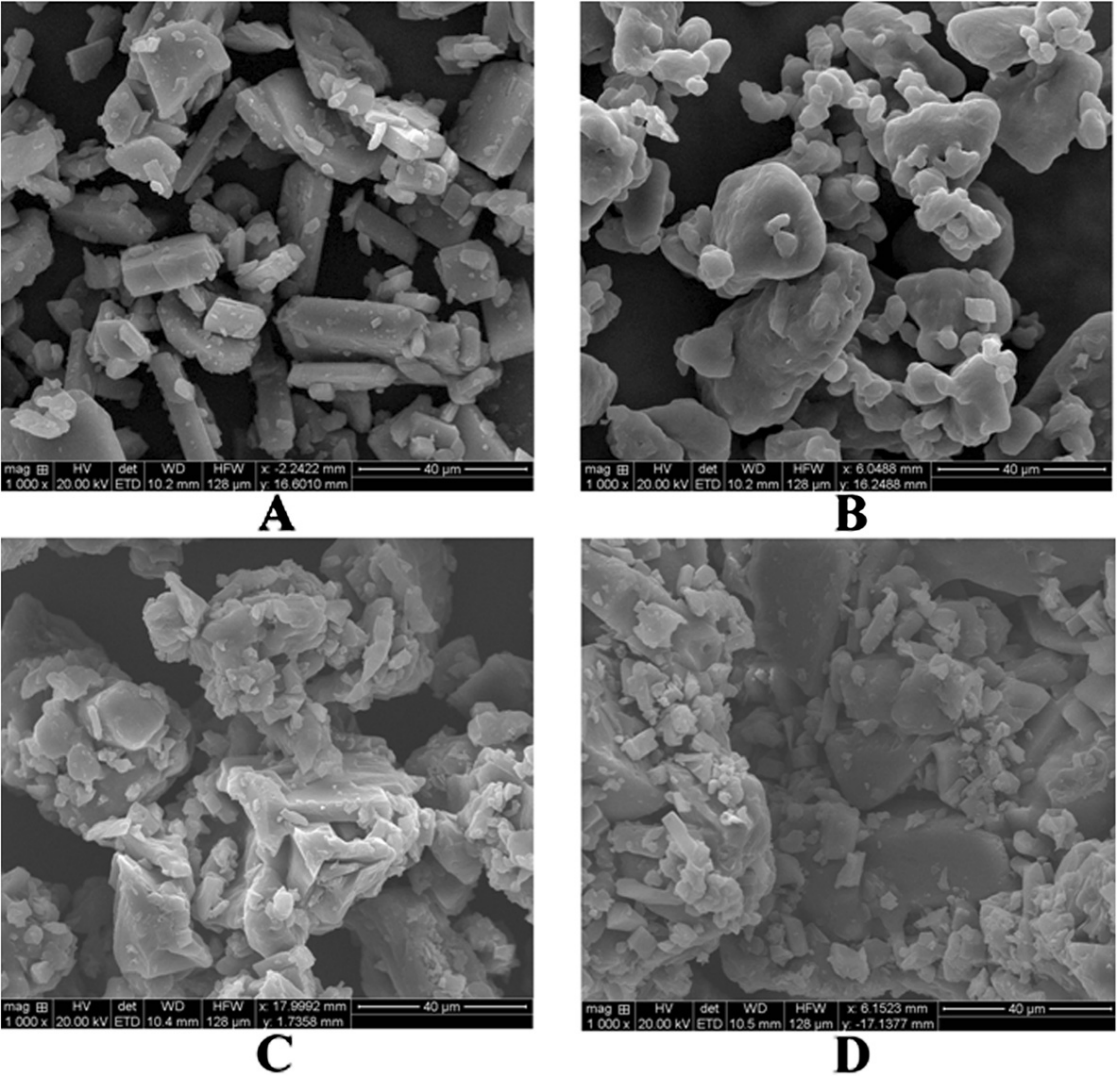
**SEM photographs of A) Indomethacin, B) SFE1815 C) S**_**6**_**D) T**_**8**_.

Figure 7, demonstrates the surface morphology of pure indomethacin as crystalline in nature. Surface morphology of S_6_ and T_8_ indicated that the individual surface properties of drug were changed during the compression process and surfactant might have been adsorbed on to the drug during the preparation of SD. The appearance of the solid dispersion was homogenous, with partial loss of drug crystallinity and reduction in particle size, which may be reason for faster dissolution of the drug and which was further confirmed by DSC and XRD studies.

## Conclusion

This study clearly shows that SD of indomethacin with SFE 1815 employing solvent evaporation technique improves their dissolution rates. Solvent evaporation techniques used in the present study requires very few quantity of organic solvent and absence of specialized equipment. Mechanisms involved are solubilisation and improved wetting of the drug [24] in the SFE 1815 rich micro-environment formed at the surface of drug crystals after dissolution of the polymer [25]. Solid dispersions formulated with SFE 1815 improved the dissolution rate compared with physical mixtures and SD formulated using PVP and PEG 4000 at the same concentrations. As SFE 1815 belongs to third generation by virtue of their efficiency to increase the surface area and wide range of HLB, these are more efficient than other commonly used carriers to increase the solubility and dissolution. There are no reports to date on the usage of SFE 1815 as carriers. The crystallinity of the drugs was reduced in all solid dispersions which were evident from the XRD graphs and decreased intensity of the peaks from DSC thermograms. The same enhanced release was observed from the SD formulated as tablet dosage form with DCL21 as diluent.

## Competing interests

The author(s) declare that they have no competing interests.

## Authors’ contributions

SAS: The main author involved in the literature survey, compilation, acquisition of data, planning design and carrying out the research work, interpretation of data along with review of intellectual content and drafting of the final manuscript titled “Investigation on *in vitro* dissolution rate enhancement of indomethacin by using a novel carrier sucrose fatty acid ester”. MVS: Co- research scholar who was involved in design of experimental formulas, carrying out the bench work for formulation of the solid dispersions and carrying out the dissolution studies. NSR: Co-research scholar involved in the analytical method development and interpretation of the FTIR studies. VR: Senior research scholar involved in the study, procurement of the novel third generation carrier sucrose fatty acid ester along with the softwares required for interpretation and drawing of dissolution profiles. KVRM: Research guide, who gave valuable suggestions in the design of experimental formulas, interpretation of the dissolution data along with the carrier role in the enhancement, critical review of the manuscript for intellectual content, vital and crucial review and approval of the final manuscript to be published. He also granted me permission to carry out research activities along with use of the equipment in the laboratory. All the above authors read and approved the final manuscript.
